# Lateral Cutaneous Branch of the Intercostal Nerve as a Donor Site for Nerve Reconstruction Following Enucleation of an Upper Extremity Schwannoma

**DOI:** 10.1002/ccr3.72068

**Published:** 2026-03-11

**Authors:** Erika Koyama, Yoshinori Suzuki, Shuji Yamashita

**Affiliations:** ^1^ Department of Plastic and Reconstructive Surgery Kawasaki Medical School Okayama Japan

**Keywords:** lateral cutaneous branch of the intercostal nerve, median nerve, nerve graft, nerve reconstruction, schwannoma

## Abstract

The lateral cutaneous branch of the intercostal nerve is a reliable donor for upper extremity nerve reconstruction after schwannoma enucleation, offering easy anatomical access and potential for functional recovery.

## Graphical abstract

Fascicular defect after median nerve schwannoma enucleation reconstructed with a graft from the lateral cutaneous branch of the intercostal nerve.

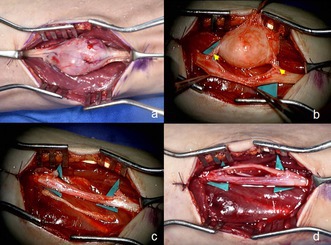

## 1 Introduction

Schwannomas are benign nerve sheath tumors consisting of differentiated Schwann cells. They can occur anywhere in the body, grow slowly, and are often associated with degenerative changes, including cyst formation, fibrosis, and calcification after bleeding [1, 2, 3]. As schwannomas are usually well‐encapsulated—except for the rare plexiform variant—enucleation of the tumor is the standard surgical procedure for treatment and yields minimal neural damage.

However, even with meticulous surgical techniques, neurological complications often occur after surgical treatment for schwannomas. The incidence of surgical complications reported in previous studies ranges from 46.5% to 73.2% [4, 5, 6, 7]. Nerve reconstruction may be performed to resolve postoperative neuropathy associated with tumor resection.

The sural nerve is most commonly used to harvest tissue for nerve grafting for upper extremity nerve defects following enucleation of schwannomas [1, 3, 8]. However, the lateral cutaneous branch of the intercostal nerve (LCB nerve) is an alternative source of graft tissue: it can be harvested from the axilla, where the donor scar is inconspicuous because of axillary hair, and is in the same surgical field in upper extremity surgery.

Here, we present the case of a patient who underwent nerve gap reconstruction using the LCB nerve following enucleation of a histologically confirmed schwannoma of the forearm. We also evaluate the sensory recovery outcomes of the affected fingers and donor site.

## 2 Case History/Examination

A 42‐year‐old man presented with a 3 × 3 × 2.7‐cm tumor mass in the left forearm. He had a positive Tinel's sign on the index and middle fingers.

## 3 Differential Diagnosis, Investigations and Treatment

**FIGURE 1:**
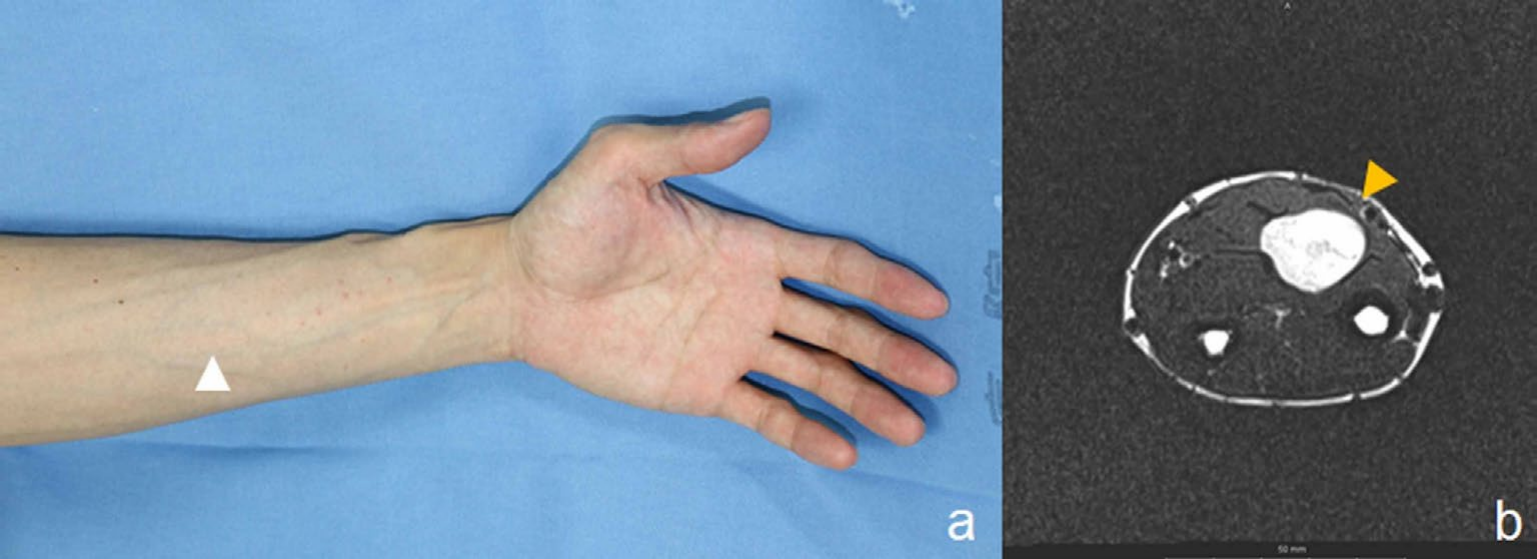
(a) The schwannoma on the flexor side of the forearm. (b) The magnetic resonance image shows that the schwannoma involves the median nerve in the forearm (arrowhead).

Differential diagnosis included schwannoma, neurofibroma, and malignant peripheral nerve sheath tumor. Magnetic resonance imaging showed a schwannoma in the median nerve (Figure 1).

**FIGURE 2:**
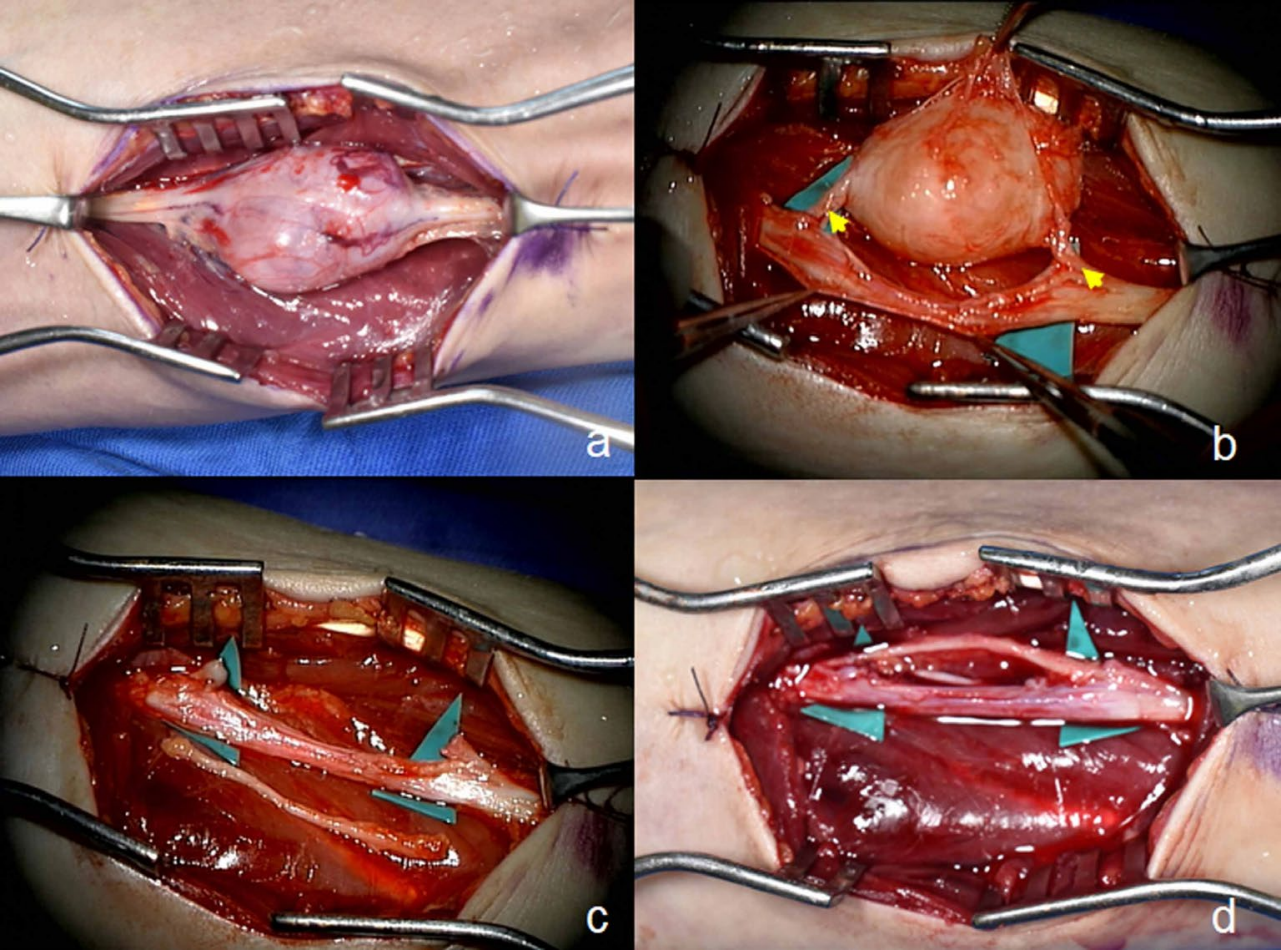
Operative photographs showing enucleation of the schwannoma and nerve reconstruction of the nerve fascicle defect. (a) The schwannoma in the median nerve is visible inside the epineurial layer. (b) The nerve fascicle (arrowhead) penetrating the schwannoma is resected. (c) The transected nerve fascicle (yellow arrow), the resulting nerve fascicle defect (blue arrow), and the reconstructed nerve graft (white arrow). (d) Reconstruction of the nerve fascicle defect using the lateral cutaneous branch of the intercostal nerve (arrowhead).

All procedures were performed under a surgical microscope with a pneumatic tourniquet. An incision was made on the flexor side of the middle forearm to expose the mass between the flexor digitorum profundus and flexor digitorum superficialis muscles. A longitudinal incision was carefully made in the epineurium to avoid damaging the fascicles, and uninvolved fascicles were dissected and retracted extracapsularly. The tumor was enucleated by cutting a single fascicle penetrating it (Figure 2a–c).

**FIGURE 3:**
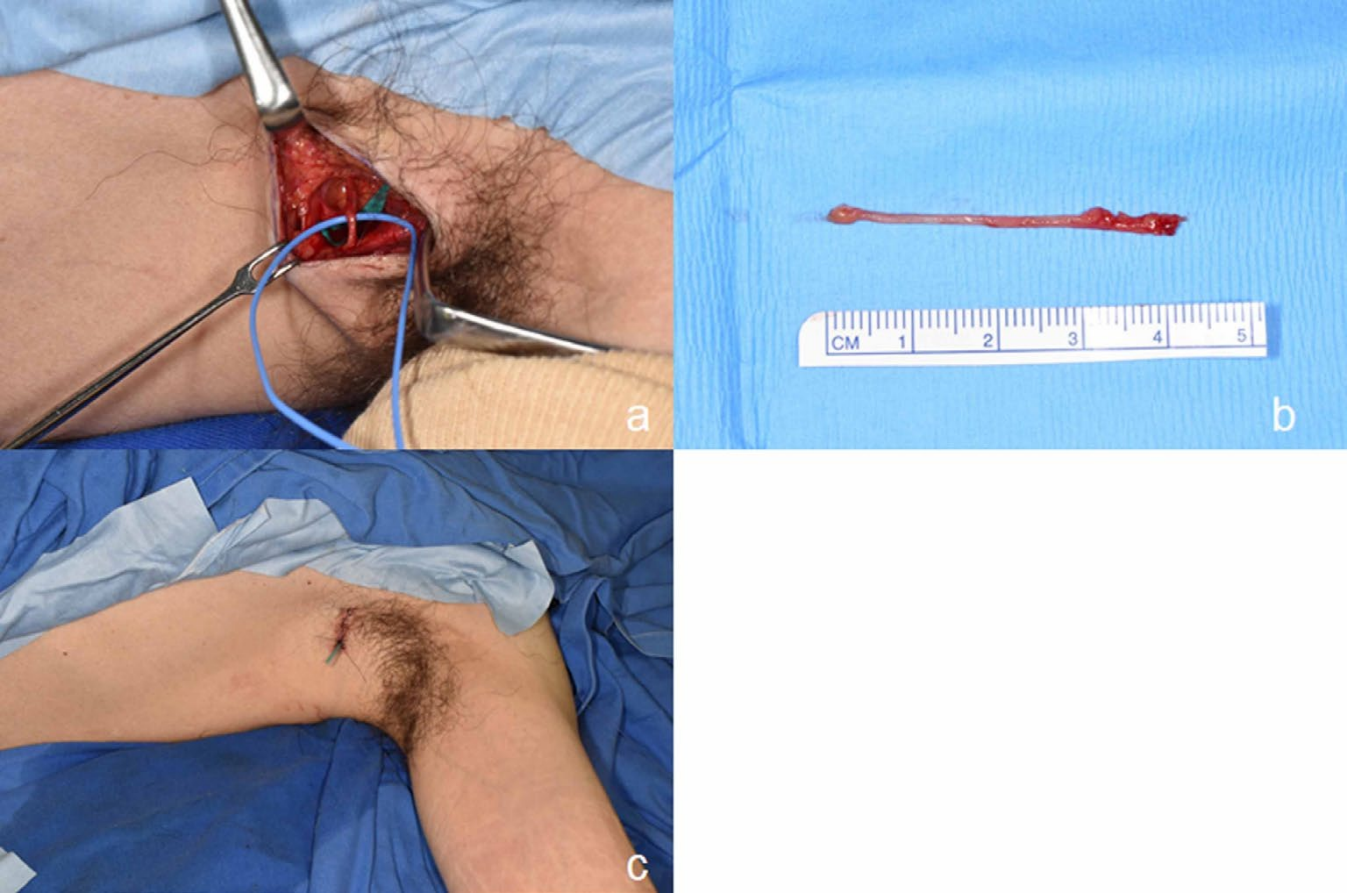
Harvest of the lateral cutaneous branch of the intercostal nerve using the axillary approach. (a) The nerve graft is harvested from the ipsilateral axilla. (b) The 4‐cm nerve graft. (c) Closure of the donor site.

To reconstruct the nerve fascicles after tumor enucleation, the LCB nerve was harvested from the axilla ipsilateral to the affected limb (Figure 3). A skin incision was made along the relaxed skin tension line within the axillary hair. A 4‐cm nerve fascicle defect was reconstructed with 10‐0 nylon using the nerve graft (Figure 2d), following which skin closure was performed.

## 4 Conclusion and Results (Outcome and Follow‐Up)

**FIGURE 4:**
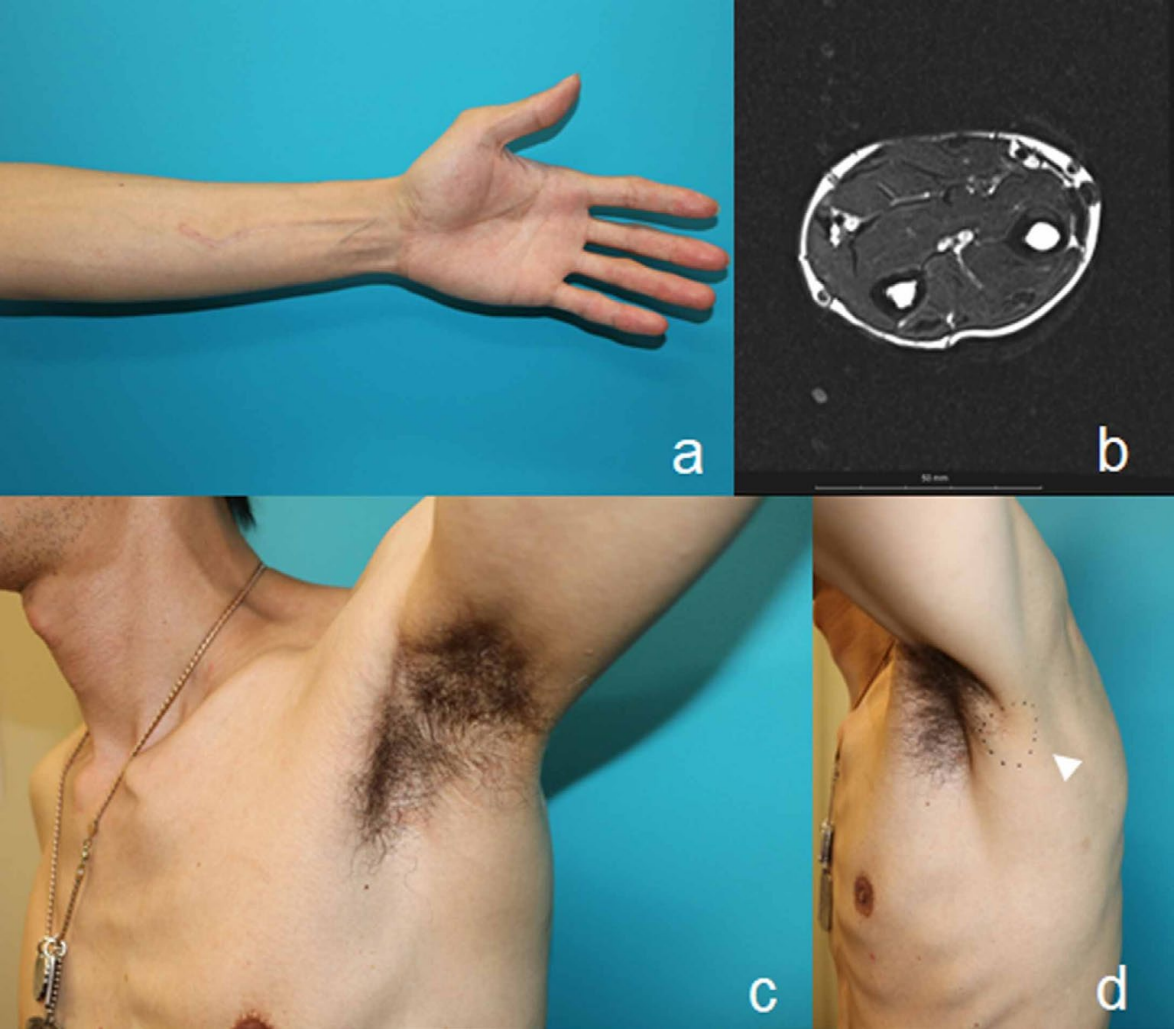
The forearm where the schwannoma was removed and the donor site, 13 months after surgery. (a) Postoperative hypesthesia of the index and middle fingers gradually improved. (b) Magnetic resonance imaging shows no recurrence of the tumor. (c) Scarring at the donor site is acceptable. (d) The site of the postoperative hypesthesia of the donor site is gradually reduced in size, leaving a 3 × 4 cm area of hypoesthesia behind the axilla (arrowhead).

Objective sensory and motor function assessments were performed for the recipient median nerve. Sensory function was evaluated using the Semmes–Weinstein monofilament (SWM) test. Preoperatively, SWM scores were normal in both the index and middle fingers (1.55 and 1.55, respectively). On postoperative day 5, sensory disturbance was observed, with SWM scores worsening to 3.66 in the index finger and 3.88 in the middle finger. Sensory function gradually improved, and at the final follow‐up 13 months after surgery, SWM scores recovered to 1.67 in the index finger and 1.55 in the middle finger. Motor function of the median nerve was assessed clinically using the Medical Research Council (MRC) motor scale. No motor dysfunction was present preoperatively, and no new postoperative motor deficit was observed during the follow‐up period (MRC grade M5 both pre‐ and post‐operatively). The scar of the donor site was hidden by the axillary hair and was esthetically acceptable because of the incision line along the relaxed skin tension line. Hypesthesia of the donor site was noted in the early postoperative period, but it gradually reduced in size to an area of 3 × 4 cm (Semmes–Weinstein monofilament test score, 3.22) behind the axilla. There was no recurrence of the tumor 13 months after surgery (Figure 4).

## 5 Discussion

The incidence of postoperative new neurological deficits with schwannoma resection is reported to be 46.5%–73.2% [4, 5, 6, 7]. Moreover, complete recovery of sensation is achieved in only 25% of patients who undergo enucleation of schwannomas without nerve reconstruction [5]. These high rates of new neurological deficits and poor sensory recovery are believed to be due to the transection of fascicles that run through the tumor. In the present case, objective functional evaluation using standardized sensory and motor assessment scales demonstrated near‐complete recovery of sensory function without any postoperative motor deficit, supporting the clinical effectiveness of fascicular nerve reconstruction using the lateral cutaneous branch of the intercostal nerve.

Recommendations have been reported for nerve reconstruction for fascicle defects [1, 2, 3, 4, 6, 8, 9]. Ozdemir et al. noted that nerve reconstruction should be attempted when tumor resection is accompanied by nerve transection [2]. Tang et al. used the sural nerve for nerve reconstruction in patients as some patients who did not undergo nerve reconstruction developed sensory impairment [3]. However, the necessity of nerve reconstruction after schwannoma enucleation remains controversial. Some authors have reported that transection of nerve fascicles penetrating the tumor does not necessarily result in postoperative neurological deficits, suggesting that reconstruction may be unnecessary in certain cases [10, 11]. In the present case, nerve reconstruction was performed based on the intraoperative finding of a functioning nerve fascicle penetrating the tumor that required transection to achieve complete enucleation. Given the documented high risk of postoperative sensory deficits associated with fascicular transection, reconstruction was undertaken with the aim of minimizing postoperative neurological impairment. This decision was further supported by the availability of a suitable donor nerve that could be harvested with minimal morbidity from the same operative field. This case illustrates that selective nerve reconstruction, guided by intraoperative findings and informed by existing evidence on postoperative neurological outcomes, may contribute to improved functional recovery following schwannoma enucleation.

The sural nerve has been the site used for harvesting nerve tissue for reconstruction with schwannoma resections [1, 3, 8]. However, chronic postoperative donor‐site complications following nerve reconstruction, such as sensory deficits, chronic pain, sensory symptoms, wound infections, wound complications, other postoperative complications, and complications impacting daily life that may negatively affect quality of life, have been reported [12].

The LCB nerves, which are derived from the 12 pairs of thoracic spinal nerves that divide into an anterior and a posterior branch, supply the overlying skin of the lateral thoracic region. Although LCB nerves have been used as sensate flaps that can be harvested from the lateral thoracic region or to be used to achieve perception of a reconstructed breast, they have not been used for nerve grafting [13, 14, 15]. Because transection of the nerve fascicle with enucleation was necessary for our patient, we used the LCB nerve for graft tissue. Use of the LCB nerves has the advantages of harvesting from an inconspicuous donor site that has minimal neurological deficit symptoms, and of the donor site being in the same surgical field in upper limb surgery. Moreover, the diameter of the LCB nerve is appropriate for digital nerves or nerve fascicle nerves in upper extremity surgery. However, the length and diameter of LCB nerves are limited. At most, a 6‐cm length can be harvested unless intramuscular dissection is performed. Therefore, the use of sural nerves is indicated when longer nerve defects or thicker nerves are required for reconstruction.

The prognosis in the present case was favorable. Sensory function of the affected fingers recovered to near‐normal levels, no postoperative motor deficit was observed, and there was no evidence of tumor recurrence at 13 months of follow‐up. The main clinical lesson from this case is that the lateral cutaneous branch of the intercostal nerve can serve as a practical and reliable donor nerve for short fascicular defects following schwannoma enucleation in the upper extremity. This donor nerve offers minimal donor‐site morbidity, an inconspicuous scar, and the advantage of being harvested within the same operative field. Although the indication is limited to short nerve gaps, this approach may be particularly useful when the goal is to minimize postoperative neurological deficits while avoiding the morbidity associated with sural nerve harvest.

## Author Contributions

**Erika Koyama:** writing – original draft. **Yoshinori Suzuki:** writing – original draft. **Shuji Yamashita:** writing – original draft.

## Funding

The authors have nothing to report.

## Disclosure

The authors have nothing to report.

## Ethics Statement

Approval was obtained from the internal review board.

## Consent

The study was conducted per the Declaration of Helsinki. Written informed consent was obtained from the study participant, including consent to participate and publish the findings.

## Conflicts of Interest

The authors declare no conflicts of interest.

## Data Availability

The data that support the findings of this study are available from the corresponding author upon reasonable request.
